# Supplementary material for: Polymorphism of pyrene on compression to 35 GPa in a diamond anvil cell
Source: Commun Chem. 2024 Sep 17;7:209. doi: 10.1038/s42004-024-01294-0 (PMC11405754; doi:10.1038/s42004-024-01294-0)
Supplement: Supplementary file 2 — Supplemental Information [file 42004_2024_1294_MOESM2_ESM.pdf]

Supplementary Information

Polymorphism of pyrene on compression to 35 GPa in a diamond anvil cell

Wenju Zhou[1*], Yuqing Yin[2], Dominique Laniel[3], Andrey Aslandukov[1,4], Elena Bykova[5], Anna Pakhomova[6], Michael Hanfland[6], Tomasz Poreba[6], Mohamed Mezouar[6], Leonid Dubrovinsky[4], Natalia Dubrovinskaia[1,2*]

[1]Material Physics and Technology at Extreme Conditions, Laboratory of Crystallography, University of Bayreuth, Bayreuth, Germany

[2]Department of Physics, Chemistry and Biology (IFM), Linköping University, Linköping, Sweden

[3]Centre for Science at Extreme Conditions and School of Physics and Astronomy,

University of Edinburgh, Edinburgh, UK

[4]Bayerisches Geoinstitut, University of Bayreuth, Bayreuth, Germany

[5]Institut für Geowissenschaften, Goethe-Universität Frankfurt, Frankfurt am Main, Germany

[6]European Synchrotron Radiation Facility, Grenoble, France

e-mail: Wenju.Zhou@uni-bayreuth.de; Natalia.Dubrovinskaia@uni-bayreuth.de

Supplementary Table 1. Summary of the high-pressure experiments conducted in this work.

<table>
<tr><th>DAC number</th><th>DAC type</th><th>Anvils type/ culet size, μm</th><th>Starting material/ pressure transmitting medium</th><th>Beamline/XRD wavelength, Å</th><th>Result</th><th>Pressure, GPa</th></tr>
<tr><td>1</td><td>BX90</td><td>Boehler-Almax 500</td><td>Pyrene-I/ No medium</td><td>ID27 ESRF, 0.3738</td><td>Pyrene-I</td><td>ambient</td></tr>
<tr><td>2</td><td>BX90</td><td>Boehler-Almax 250</td><td>Pyrene-I/ Neon</td><td>ID15B ESRF, 0.4100</td><td>Pyrene-V</td><td>2.5
13.5
17.0</td></tr>
<tr><td rowspan="3">3</td><td rowspan="3">Membrane-type</td><td rowspan="3">Boehler-Almax 250</td><td rowspan="3">Pyrene-I/ Helium</td><td rowspan="3">ID27 ESRF, 0.3738</td><td>Pyrene-II</td><td>0.7
1.4</td></tr>
<tr><td>Pyrene-IV</td><td>2.7
4.3</td></tr>
<tr><td>Pyrene-V</td><td>7.3
9.5
15.4
20.2
29.8
35.5</td></tr>
<tr><td>4</td><td>BX90</td><td>Boehler-Almax 250</td><td>Pyrene-I/ KCl</td><td>ID15B ESRF, 0.4100</td><td>Pyrene-III
Pyrene-IV</td><td>6.5</td></tr>
</table>

Supplementary Table 2. Experimental crystallographic data for pyrene-I at ambient conditions obtained by single-crystal X-ray diffraction in this work and by neutron diffraction in ref. [14].

	Pyrene-I, this work	Pyrene-I, ref. [14]
CCDC deposition number*	2360694	1240736
Crystal data		
Chemical formula	$C_{16}H_{10}$	$C_{16}H_{10}$
M_r	202.24	202.24
Crystal system, space group	Monoclinic, $P2_1/c$	Monoclinic, $P2_1/c$
a, b, c (Å)	8.478(8), 9.2562(12), 13.655(7)	8.470(4), 9.253(5), 13.649(7)
α, β, γ (°)	90, 100.31(8), 90	90, 100.28(3), 90
V (Å^3)	1055.3(11)	1052.5(4)
Z	4	4
Density (Mg/m^3)	1.273	1.27
Wavelength (Å)	0.3738	1.025 (neutron radiation)
μ (mm^{-1})	0.031	0.155
Data collection		
Absorption correction	Multi-scan	Linear absorption coefficient
T_{min}, T_{max}	0.036, 1.00	
No. of measured, independent and observed reflections	2963, 1269, 456	3500, 1492, 1008
R_{int}	0.023	
θ_{max} (°)	18.32	34.67
Refinement		
Refinement on	F^2	F^2
$R[F^2>2\sigma(F^2)], wR(F^2), S$	0.049, 0.16, 0.92	0.034, 0.032, 0.89
Data / restraints / parameters	1269/ 0/ 145	1008/ 0/ 236
H-atom treatment	Refined by ride model	No restraints
Weighting scheme	$w = 1/[\sigma^2 (F_o^2) + (0.0539P)^2]$, where $P= (F_o^2+2F_c^2)/3$	$w = 1/\sigma^2$, where $\sigma = 1/[\sigma_c(F^2) + (1+0.02)F^2 - F]$
$\Delta\rho_{max}$, $\Delta\rho_{min}$ (e Å^{-3})	0.08, -0.08	

*CCDC number refers to the supplementary crystallographic data for this paper. These data can be obtained free of charge from The Cambridge Crystallographic Data Centre via www.ccdc.cam.ac.uk/structures

Supplementary Table 3. Experimental crystallographic data for pyrene-II obtained by single-crystal X-ray diffraction at room temperature and two different pressures in this work and at 93 K at ambient pressure in ref. [10].

	Pyrene-II at 0.7 GPa	Pyrene-II at 1.4 GPa	Pyrene-II at 93 K, ref. [10]
CCDC deposition number	2360720	2360721	118728
Crystal data			
Chemical formula	$C_{16}H_{10}$	$C_{16}H_{10}$	$C_{16}H_{10}$
M_r	202.24	202.24	202.24
Crystal system, space group	Monoclinic, $P2_1/c$	Monoclinic, $P2_1/c$	Monoclinic, $P2_1/c$
a, b, c (Å)	8.1431(12), 9.8639(7), 12.1136(4)	8.0322(9), 9.7422(6), 11.8112(4)	8.260(4), 10.020(4), 12.358(6)
α, β, γ (°)	90, 96.484(7), 90	90, 96.074(7), 90	90, 96.48(4), 90
V (Å^3)	966.77(16)	919.05(12)	1016.3(8)
Z	4	4	4
Density (Mg/m^3)	1.389	1.462	1.322
Wavelength (Å)	0.3738	0.3738	0.71069
μ (mm^{-1})	0.035	0.035	0.075
Data collection			
Absorption correction	Multi-scan	Multi-scan	Multi-scan
T_{min}, T_{max}	0.20, 1.00	0.24, 1.00	
No. of measured, independent and observed reflections	1864, 1390, 830	2380, 1414, 832	10829, 2582, 1519
R_{int}	0.018	0.031	0.051
θ_{max} (°)	20.05	18.13	29.5
Refinement			
Refinement on	F^2	F^2	F^2
$R[F^2>2\sigma(F^2)]$,$wR(F^2)$, S	0.068, 0.208, 1.04	0.061, 0.182, 1.01	0.052, 0.091, 1.01
Data / restraints / parameters	1390/ 0/ 172	1414/ 0/ 175	1390/ 0/ 172
H-atom treatment	Refined by ride model	Refined by ride model	Refined by ride model
Weighting scheme	$w = 1/[\sigma^2 (F_o^2) + (0.1448P)^2]$, where $P = (F_o^2 + 2F_c^2)/3$	$w = 1/[\sigma^2(F_o^2) + (0.1028P)^2]$, where $P = (F_o^2 + 2F_c^2)/3$	$w = 1/[\sigma^2(F_o^2) + (0.045P)^2 + 0.15P]$, where $P = (F_o^2 + 2F_c^2)/3$
$\Delta\rho_{max}$, $\Delta\rho_{min}$ (e Å^{-3})	0.17, -0.17	0.19, -0.17	0.34, -0.26

Supplementary Table 4. Experimental crystallographic data for pyrene-IV obtained by single-crystal X-ray diffraction at room temperature in this work.

	Pyrene-IV at 2.7 GPa	Pyrene-IV at 4.3 GPa
CCDC deposition number	2360722	2360723
Crystal data		
Chemical formula	$C_{16}H_{10}$	$C_{16}H_{10}$
M_r	202.24	202.24
Crystal system, space group	Triclinic, *P*-1	Triclinic, *P*-1
a, b, c (Å)	7.593(3), 10.223(3), 11.192(2)	7.477(2), 10.070(2), 10.879(3)
α, β, γ (°)	92.536(19), 95.04(2), 91.21(3)	92.434(19), 94.73(2), 90.86(2)
V ($Å^3$)	864.2(5)	815.5(4)
Z	4	4
Density (Mg/m^3)	1.554	1.647
Wavelength (Å)	0.3738	0.3738
μ (mm^{-1})	0.038	0.040
Data collection		
Absorption correction	Multi-scan	Multi-scan
T_{min}, T_{max}	0.34, 1.00	0.18, 1.00
No. of measured, independent and observed reflections	657, 604, 415	1378, 1036, 558
R_{int}	0.015	0.034
θ_{max} (°)	14.43	14.83
Refinement		
Refinement on	F^2	F^2
$R[F^2>2\sigma(F^2)]$,$wR(F^2)$, S	0.098, 0.305, 1.07	0.134, 0.393, 1.40
Data / restraints / parameters	604/ 11/ 129	1036/ 0/ 129
H-atom treatment	Refined by ride model	Refined by ride model
Weighting scheme	$w = 1/[\sigma^2(F_o^2) + (0.1836P)^2 + 1.7101P]$, where $P = (F_o^2 + 2F_c^2)/3$	$w = 1/[\sigma^2 (F_o^2) + (0.2P)^2]$, where $P= (F_o^2+2F_c^2)/3$
$\Delta\rho_{max}$, $\Delta\rho_{min}$ (e $Å^{-3}$)	0.18, -0.17	0.51, -0.38

Supplementary Table 5. Experimental crystallographic data for pyrene-V obtained by single-crystal X-ray diffraction at room temperature in this work.

	Pyrene-V at 7.3 GPa	Pyrene-V at 9.5 GPa	Pyrene-V at 15.4 GPa
CCDC deposition number	2360724	2360725	2360726
Crystal data			
Chemical formula	$C_{16}H_{10}$	$C_{16}H_{10}$	$C_{16}H_{10}$
M_r	202.24	202.24	202.24
Crystal system, space group	Monoclinic, $P2_1/c$	Monoclinic, $P2_1/c$	Monoclinic, $P2_1/c$
a, b, c (Å)	7.450(5), 6.4503(12), 16.096(2)	7.345(4), 6.2802(8), 15.9325(19)	7.2292(3), 6.0333(14), 15.7590(6)
α, β, γ (°)	90, 100.65(3), 90	90, 100.57(3), 90	90, 100.516(5), 90
V (Å^3)	760.1(5)	722.5(4)	675.80(16)
Z	4	4	4
Density (Mg/m^3)	1.767	1.859	1.988
Wavelength (Å)	0.3738	0.3738	0.3738
μ (mm^{-1})	0.043	0.045	0.048
Data collection			
Absorption correction	Multi-scan	Multi-scan	Multi-scan
T_{min}, T_{max}	0.21, 1.00	0.15, 1.00	0.23, 1.00
No. of measured, independent and observed reflections	592, 488, 274	738, 595, 295	1021, 776, 632
R_{int}	0.063	0.075	0.051
θ_{max} (°)	13.50	14.06	29.5
Refinement			
Refinement on	F^2	F^2	F^2
$R[F^2>2\sigma(F^2)]$,$wR(F^2)$, S	0.087, 0.222, 0.99	0.12, 0.31, 1.16	0.078, 0.023, 1.12
Data / restraints / parameters	488/ 0/ 66	595/ 0/ 66	776/ 0/ 145
H-atom treatment	Refined by ride model	Refined by ride model	Refined by ride model
Weighting scheme	$w = 1/[\sigma^2 (F_o^2) + (0.729P)^2]$, where $P = (F_o^2 + 2F_c^2)/3$	$w = 1/[\sigma^2(F_o^2) + (0.200P)^2]$, where $P = (F_o^2 + 2F_c^2)/3$	$w = 1/[\sigma^2(F_o^2) + (0.198P)^2]$, where $P = (F_o^2 + 2F_c^2)/3$
$\Delta\rho_{max}$, $\Delta\rho_{min}$ (e Å^{-3})	0.20, -0.20	0.19, -0.17	0.53, -0.33

Supplementary Table 5. (continuation)

Pyrene-V at 20.2 GPa	Pyrene-V at 25.2 GPa	Pyrene-V at 29.8 GPa	Pyrene-V at 35.5 GPa
2360727	2360728	2360729	2360730
$C_{16}H_{10}$	$C_{16}H_{10}$	$C_{16}H_{10}$	$C_{16}H_{10}$
202.24	202.24	202.24	202.24
Monoclinic, $P2_1/c$	Monoclinic, $P2_1/c$	Monoclinic, $P2_1/c$	Monoclinic, $P2_1/c$
7.1550(3), 5.8939(13), 15.6277(6)	7.0634(3), 5.7352(14), 15.4503(6)	7.0174(5), 5.645(3), 15.3477(13)	6.9711(7), 5.541(4), 15.2471(16)
90, 100.467(5), 90	90, 100.395(5), 90	90, 100.363(8), 90	90, 100.349(10), 90
648.07(15)	615.62(15)	598.0(3)	579.4(4)
4	4	4	4
2.073	2.182	2.246	2.318
0.3738	0.3738	0.3738	0.3738
0.050	0.053	0.054	0.056
Multi-scan	Multi-scan	Multi-scan	Multi-scan
0.15, 1.00	0.01, 1.00	0.04, 1.00	0.34, 1.00
1004, 835, 654	990, 808, 630	760, 684, 521	560, 531, 403
0.016	0.018	0.017	0.060
19.95	20.25	20.41	19.84
F^2	F^2	F^2	F^2
0.076, 0.231, 1.08	0.136, 0.338, 1.60	0.136, 0.345, 1.61	0.166, 0.392, 1.80
835/ 0/ 145	808/ 96/ 145	684/ 96/ 145	531/ 96/ 145
Refined by ride model	Refined by ride model	Refined by ride model	Refined by ride model
$w = 1/[\sigma^2(F_o^2) + (0.181P)^2 + 0.285P]$, where $P = (F_o^2 + 2F_c^2)/3$	$w = 1/[\sigma^2 (F_o^2) + (0.2P)^2]$, where $P = (F_o^2 + 2F_c^2)/3$	$w = 1/[\sigma^2(F_o^2) + (0.2P)^2]$, where $P = (F_o^2 + 2F_c^2)/3$	$w = 1/[\sigma^2(F_o^2) + (0.2P)^2]$, where $P = (F_o^2 + 2F_c^2)/3$
0.46, -0.28	0.54, -0.59	0.41, -0.42	0.51, -0.54

Supplementary Table 6. Unit cell volume per formula unit for pyrene polymorphs up to 35.5 GPa in this work and in ref. [6].

Polymorph/ pressure transmitting medium	Pressure, GPa	Volume per formula unit, Å^3
Pyrene-I/ No medium	0	263.83(3)
Pyrene-II/ He	0.7	241.69(4)
Pyrene-II/ He	1.4	229.76(3)
Pyrene-III/ dichloromethane [6]	0.3	249(3)
Pyrene-III/ dichloromethane [6]	0.5	238.91(9)
Pyrene-IV/ He	2.7	216.05(16)
Pyrene-IV/ He	4.3	203.88(10)
Pyrene-V/ He	7.3	190.02(16)
Pyrene-V/ He	9.5	180.63(10)
Pyrene-V/ He	15.4	169.20(4)
Pyrene-V/ He	20.2	162.02(4)
Pyrene-V/ He	25.2	153.90(4)
Pyrene-V/ He	29.8	149.50(9)
Pyrene-V/ He	35.5	144.85(10)
Pyrene-V/ Ne	2.5	213.92(4)
Pyrene-V/ Ne	13.5	174.45(15)
Pyrene-V/ Ne	17	167.08(8)

Supplementary Table 7. DFT-calculated unit cell volume per formula unit of pyrene polymorphs up to 36 GPa.

Polymorph	Pressure (GPa)	Volume per formula unit (Å^3)
Pyrene-I	0	247.71
Pyrene-II	1	229.64
Pyrene-II	2	219.12
Pyrene-IV	3	209.86
Pyrene-IV	4	203.54
Pyrene-V	6	191.04
Pyrene-V	9	180.58
Pyrene-V	13	170.96
Pyrene-V	17	163.82
Pyrene-V	21	158.07
Pyrene-V	24	154.41
Pyrene-V	27	151.19
Pyrene-V	30	148.28
Pyrene-V	33	145.80
Pyrene-V	36	143.37

Supplementary Table 8. Lattice parameters of pyrene polymorphs up to 35.5 GPa determined in this work and in ref. [6]

Polymorph/ pressure transmitting medium	Pressure, GPa	*a*, Å	*b*, Å	*c*, Å	*α*, °	*β*, °	*γ*, °
Pyrene-I/ no medium	0	8.478(8)	9.2652(12)	13.655(7)	90	100.31(8)	90
Pyrene-II/ He	0.7	8.1431(12)	9.8639(7)	12.1136(4)	90	96.484(7)	90
Pyrene-II/ He	1.4	8.0322(9)	9.7422(6)	11.8112(4)	90	96.074(7)	90
Pyrene-III/ dichloromethane [6]	0.3	8.65(7)	3.852(3)	15.35(9)	90	103.3(4)	90
Pyrene-III/ dichloromethane [6]	0.5	8.3341(16)	3.8375(5)	15.309(4)	90	102.606(19)	90
Pyrene-IV/ He	2.7	7.593(3)	10.223(3)	11.192(2)	92.536(19)	95.04(2)	91.21(3)
Pyrene-IV/ He	4.3	7.477(2)	10.070(2)	10.879(3)	92.434(19)	94.73(2)	90.86(2)
Pyrene-V/ He	7.3	7.450(5)	6.4503(12)	16.096(2)	90	100.65(3)	90
Pyrene-V/ He	9.5	7.345(4)	6.2802(8)	15.9325(19)	90	100.57(3)	90
Pyrene-V/ He	15.4	7.2292(3)	6.0333(14)	15.7590(6)	90	100.516(5)	90
Pyrene-V/ He	20.2	7.1550(3)	5.8939(13)	15.6277(6)	90	100.467(5)	90
Pyrene-V/ He	25.2	7.0634(3)	5.7352(14)	15.4503(6)	90	100.395(5)	90
Pyrene-V/ He	29.8	7.0174(5)	5.645(3)	15.3477(13)	90	100.363(8)	90
Pyrene-V/ He	35.5	6.9711(7)	5.541(4)	15.2471(16)	90	100.349(10)	90
Pyrene-V/ Ne	2.5	7.6733(13)	6.9186(3)	16.4075(9)	90	100.776(9)	90
Pyrene-V/ Ne	13.5	7.2481(7)	6.184(5)	15.8236(16)	90	100.331(12)	90
Pyrene-V/ Ne	17	7.1839(5)	6.034(3)	15.6713(8)	90	100.294(7)	90

Supplementary Table 9a. DFT-calculated crystallographic data for pyrene-I at ambient pressure.

Pyrene-I	
Chemical formula	$C_{16}H_{10}$
M_r	202.24
Crystal system, space group	Monoclinic, $P2_1/c$
a, b, c (Å)	8.309, 9.087, 13.358
α, β, γ (°)	90, 100.739, 90
V (Å^3)	990.849
Z	4

Supplementary Table 9b. Theoretical atomic coordinates for pyrene-I at ambient pressure.

Label	x	y	z	Occupancy
C1	0.07549	0.53393	0.21596	1
C2	0.48589	0.24487	0.00152	1
C3	0.36086	0.69108	0.00972	1
C4	0.78127	0.27446	0.05543	1
C5	0.21669	0.67255	0.36234	1
C6	0.07256	0.63621	0.29349	1
C7	0.63265	0.10517	0.14981	1
C8	0.4844	0.14166	0.08224	1
C9	0.2219	0.46774	0.20344	1
C10	0.63026	0.00299	0.23086	1
C11	0.52322	0.4403	0.25721	1
C12	0.66558	0.47554	0.32174	1
C13	0.33318	0.07738	0.0962	1
C14	0.18851	0.11693	0.02935	1
C15	0.19092	0.2836	0.45023	1
C16	0.3378	0.22	0.43605	1
H1	0.03831	0.00568	0.33541	1
H2	0.35957	0.61433	0.07224	1
H3	0.10308	0.82326	0.45523	1
H4	0.04235	0.18885	0.19866	1
H5	0.22367	0.38849	0.14215	1
H6	0.52383	0.3627	0.19519	1
H7	0.21897	0.57444	0.68816	1
H8	0.07324	0.06924	0.04095	1
H9	0.07701	0.25632	0.39874	1
H10	0.33987	0.1425	0.37386	1

Supplementary Table 10a. Theoretical crystallographic data for pyrene-II at 1 GPa.

Pyrene-II	
Chemical formula	$C_{16}H_{10}$
M_r	202.24
Crystal system, space group	Monoclinic, $P2_1/c$
a, b, c (Å)	7.998, 9.574, 11.966
α, β, γ (°)	90, 96.346, 90
V (Å^3)	910.681
Z	4

Supplementary Table 10b. Theoretical atomic coordinates for pyrene-II at 1 GPa.

Label	x	y	z	Occupancy
C1	0.30962	0.59097	0.07593	1
C2	0.38635	0.10838	0.36213	1
C3	0.1681	0.6432	0.00903	1
C4	0.22822	0.1766	0.36633	1
C5	0.53193	0.15805	0.42938	1
C6	0.21938	0.29121	0.4424	1
C7	0.30137	0.47303	0.14866	1
C8	0.74722	0.44303	0.27549	1
C9	0.60175	0.4906	0.20978	1
C10	0.35884	0.1618	0.00748	1
C11	0.44172	0.42468	0.21219	1
C12	0.08766	0.1279	0.2966	1
C13	0.51978	0.22522	0.00254	1
C14	0.66496	0.17731	0.06809	1
C15	0.10008	0.01203	0.22748	1
C16	0.81919	0.24246	0.06119	1
H1	0.04661	0.59363	0.01295	1
H2	0.09745	0.33949	0.44895	1
H3	0.18078	0.4208	0.15157	1
H4	0.73863	0.35194	0.32924	1
H5	0.34932	0.07539	0.06555	1
H6	0.4343	0.3334	0.26549	1
H7	0.03259	0.68077	0.20165	1
H8	0.65515	0.08935	0.12482	1
H9	0.01184	0.47484	0.32405	1
H10	0.06838	0.70508	0.38821	1

Supplementary Table 11a. Theoretical crystallographic data for pyrene-III at 1 GPa.

Pyrene-III	
Chemical formula	$C_{16}H_{10}$
M_r	202.24
Crystal system, space group	Monoclinic, $P2_1/c$
a, b, c (Å)	8.174, 3.701, 14.961
α, β, γ (°)	90, 102.437, 90
V (Å^3)	441.97
Z	2

Supplementary Table 11b. Theoretical atomic coordinates for pyrene-III at 1 GPa.

Label	x	y	z	Occupancy
C1	0.11509	0.55284	0.34116	1
C2	0.74033	0.18353	0.18261	1
C3	0.58503	0.16221	0.12051	1
C4	0.43347	0.29113	0.14258	1
C5	0.2842	0.27386	0.08086	1
C6	0.27334	0.37498	0.49144	1
C7	0.12168	0.39636	0.42645	1
C8	0.57773	0.00909	0.03217	1
H1	0.00465	0.07632	0.20723	1
H2	0.74604	0.30879	0.24926	1
H3	0.43973	0.40568	0.2102	1
H4	0.17013	0.37376	0.09898	1
H5	0.00814	0.29145	0.44391	1

Supplementary Table 12a. Theoretical crystallographic data for pyrene-IV at 3 GPa.

Pyrene-IV	
Chemical formula	$C_{16}H_{10}$
M_r	202.24
Crystal system, space group	Triclinic, *P*-1
a, b, c (Å)	7.512, 10.088, 11.129
α, β, γ (°)	92.825, 94.685, 90.639
V (Å^3)	839.45
Z	4

Supplementary Table 12b. Theoretical atomic coordinates for pyrene-IV at 3 GPa.

Label	x	y	z	Occupancy
C1	0.19644	0.04043	0.8431	1
C2	0.9842	0.1761	0.06727	1
C3	0.35799	0.02253	0.25269	1
C4	0.07558	0.2823	0.01779	1
C5	0.26603	0.11258	0.17824	1
C6	0.75675	0.44389	0.22391	1
C7	0.07993	0.09029	0.1456	1
C8	0.71091	0.04542	0.0858	1
C9	0.26162	0.30325	0.05197	1
C10	0.79736	0.15496	0.0366	1
C11	0.09219	0.51309	0.70171	1
C12	0.00927	0.02103	0.81143	1
C13	0.22027	0.58724	0.43434	1
C14	0.22335	0.46698	0.36084	1
C15	0.08855	0.89151	0.26036	1
C16	0.25498	0.31264	0.64076	1
C17	0.11391	0.15262	0.49011	1
C18	0.07132	0.41608	0.30006	1
C19	0.049	0.77516	0.51422	1
C20	0.02066	0.63634	0.06175	1
C21	0.05613	0.65572	0.4406	1
C22	0.79602	0.17843	0.4166	1
C23	0.09776	0.39082	0.63251	1
C24	0.39873	0.35568	0.7226	1
C25	0.62724	0.35889	0.49937	1
C26	0.29405	0.75925	0.04173	1
C27	0.35217	0.2237	0.13103	1

C28	0.26949	0.91387	0.29319	1
C29	0.25812	0.19292	0.56625	1
C30	0.2026	0.6565	0.09044	1
C31	0.60734	0.52368	0.21164	1
C32	0.63593	0.24513	0.42462	1
H1	0.26277	0.12582	0.8091	1
H2	0.50079	0.03632	0.27554	1
H3	0.76116	0.34998	0.17206	1
H4	0.56812	0.03137	0.06485	1
H5	0.32873	0.38522	0.01298	1
H6	0.34862	0.41518	0.35465	1
H7	0.02142	0.80408	0.28795	1
H8	0.1188	0.06164	0.43389	1
H9	0.07291	0.32185	0.24888	1
H10	0.04925	0.44337	0.89927	1
H11	0.80236	0.08824	0.35931	1
H12	0.48296	0.70531	0.26773	1
H13	0.49983	0.40567	0.50877	1
H14	0.43643	0.77561	0.06551	1
H15	0.49305	0.24243	0.15823	1
H16	0.65948	0.15496	0.64856	1
H17	0.37863	0.13356	0.57089	1
H18	0.27073	0.59214	0.1542	1
H19	0.49377	0.49074	0.15016	1
H20	0.51404	0.20757	0.37446	1

Supplementary Table 13a. Theoretical crystallographic data for pyrene-V at 9 GPa.

Pyrene-V	
Chemical formula	$C_{16}H_{10}$
M_r	202.24
Crystal system, space group	Monoclinic, $P2_1/c$
a, b, c (Å)	7.339, 6.292,<br>15.898
α, β, γ (°)	90, 100.28, 90
V (Å^3)	722.3
Z	4

Supplementary Table 13b. Theoretical atomic coordinates for pyrene-V at 9 GPa.

Label	x	y	z	Occupancy
C1	0.5488	0.09966	0.2072	1
C2	0.60866	0.19715	0.13618	1
C3	0.29593	0.15517	0.05236	1
C4	0.17771	0.3368	0.47309	1
C5	0.23743	0.07336	0.12659	1
C6	0.2603	0.66186	0.00997	1
C7	0.13868	0.61776	0.21429	1
C8	0.85721	0.33081	0.06621	1
C9	0.32011	0.55156	0.22022	1
C10	0.07992	0.71854	0.28208	1
C11	0.79753	0.25463	0.14121	1
C12	0.54986	0.21857	0.4832	1
C13	0.48383	0.21695	0.05792	1
C14	0.42838	0.22356	0.40515	1
C15	0.35871	0.04612	0.20096	1
C16	0.24544	0.28216	0.40039	1
H1	0.03344	0.37812	0.46939	1
H2	0.09401	0.02762	0.12313	1
H3	0.21018	0.61583	0.06737	1
H4	0.0393	0.58768	0.15656	1
H5	0.00176	0.37226	0.0694	1
H6	0.36218	0.46155	0.16885	1
H7	0.06411	0.26195	0.22244	1
H8	0.48002	0.19093	0.34722	1
H9	0.68841	0.48152	0.24344	1
H10	0.1579	0.29429	0.33819	1

Supplementary Table 14. The enthalpy difference ΔH (eV/molecule) of the pyrene polymorphs up to 5 GPa calculated by DFT.

Pressure, GPa	Pyrene-I	Pyrene-II	Pyrene-III	Pyrene-IV	Pyrene-V
0	0	-0.0003	0.0010	0.0181	0.0514
1	0	-0.0088	-0.0465	0.0046	0.0241
2	0	-0.0178	-0.0898	-0.0121	-0.0158
3	0	-0.0253	-0.1287	-0.0283	-0.0521
4	0	-0.0338	-0.1668	-0.0474	-0.0891
5	0	-0.0451	-0.2015	-0.0686	-0.1294

Supplementary Table 15. Intermolecular distances and interplanar angles of pyrene polymorphs up to 35.5 GPa from experiments in helium pressure medium.

Polymorph	Pressure, GPa	Intermolecular distance d_1, Å	Intermolecular distance d_2, Å	Interplanar angle, °
Pyrene-I	0	3.534	3.534	83.6
Pyrene-II	0.7	3.378	3.378	75.4
Pyrene-II	1.4	3.289	3.289	73.8
Pyrene-IV	2.7	3.148	3.239	66.7
Pyrene-IV	4.3	3.075	3.157	65.1
Pyrene-V	7.3	3.013	2.948	44.9
Pyrene-V	9.5	2.944	2.885	43.7
Pyrene-V	15.4	2.861	2.772	42
Pyrene-V	20.2	2.808	2.710	41.1
Pyrene-V	25.2	2.755	2.633	40.1
Pyrene-V	29.8	2.712	2.593	40
Pyrene-V	35.5	2.656	2.560	39.5

Supplementary Note 1. Fingerprint plot and Hirshfeld surface mapped with d_{norm}

Pyrene molecules exhibit various interactions, including π ··π stacking, C-H ··π, and H ··H contacts. As depicted in **Supplementary Fig. 1a**, the π···π stacking motif is represented in the two-dimensional fingerprint plot as a green triangle region with $d_e \approx d_i$, starting at approximately 1.75 Å (indicated by Arrow 1). The letter indicates the minimal intermolecular distance in the pairs of pyrene-I molecules. This observation aligns with the intermolecular distance of ~3.5 Å based on geometrical consideration (see main text). On either side of the plot, two distinct pairs of 'wings' are observed, representing two different C-H(e)···π(i) interactions (outlined in yellow) and two different C-H(i)···π(e) interactions (outlined in purple), respectively. **Supplementary Fig. 1b** illustrates the intermolecular interactions of pyrene-I molecules within their surrounding molecular environment. The Hirshfeld surface in this figure is mapped using d_{norm}, which incorporates both de and di values. Each is normalized by the vdW (van der Waals) radius corresponding to the atoms involved in the close contact near the surface. The advantage of using d_{norm} lies in its ability to provide a more balanced and symmetrical approach to analyze the distances between atoms. This method employs a distinct red-white-blue color scheme (differentiated from the red-green-blue scheme used for de and di) to map these distances. This design allows d_{norm} to impartially identify all close contacts, where contacts shorter than the vdW distance are displayed as red dots on a surface that is primarily blue. Fig. S1b displays three types of interactions: the primary C-H(e)···π(i) interaction and the primary C-H(i)···π(e) interaction, both represented by the lower pair of wings in the fingerprint plot, as well as the H(i)···H(e) interaction. Other non-close contact interactions are not reflected in the d_{norm}.

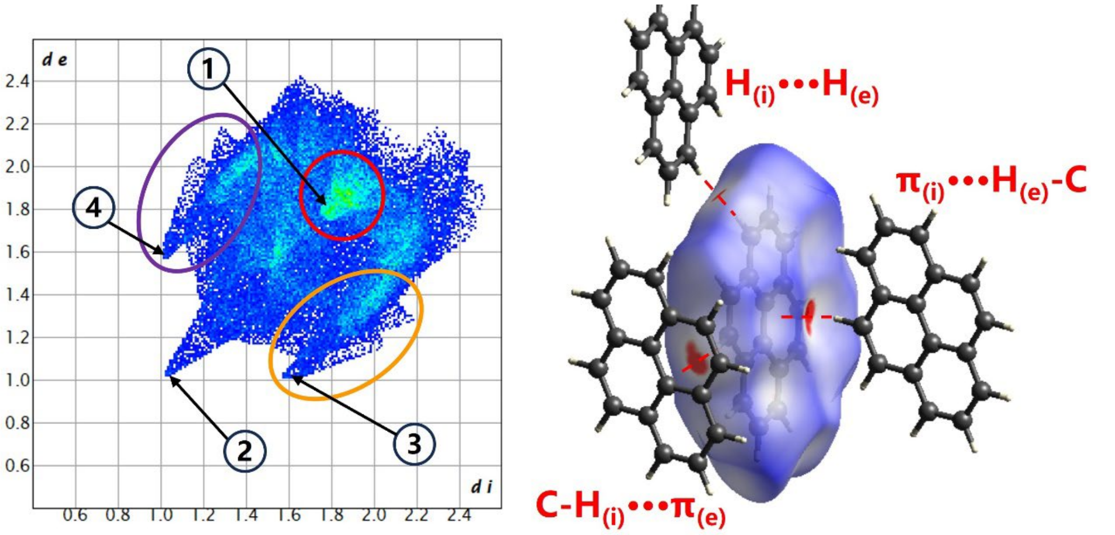

Supplementary Figure 1. Intermolecular interactions for pyrene-I. a) Fingerprint plot: the green triangle within the red circle represents the range of the π ··· π stacking interaction; the two 'wings' within the yellow oval represent the ranges of two distinct C-H(e)···π(i) interactions, and the two 'wings' in the purple oval indicate the ranges of two different C-H(i)···π(e) interactions (in the figure b to the right, only one of each kind of C-H·· π interactions is shown for the figure clarity); blue corresponds to the low frequency of occurrence of a (di, de) pair, while red points (if appear) indicate the high frequency of the surface points with that (di, de) combination; arrows numbered 1 through 4 point to the features corresponding to the shortest contacts for (1) the π···π stacking (represent the intermolecular distance), (2) H(i) ··H(e) contacts, (3) C-H(e)·· π(i) , and (4) C-H(i)·· π(e) interactions. (b) Hirshfeld surface mapped with d_{norm} from -0.12 (red) to 0 (white) to 1.46 (blue).

a)

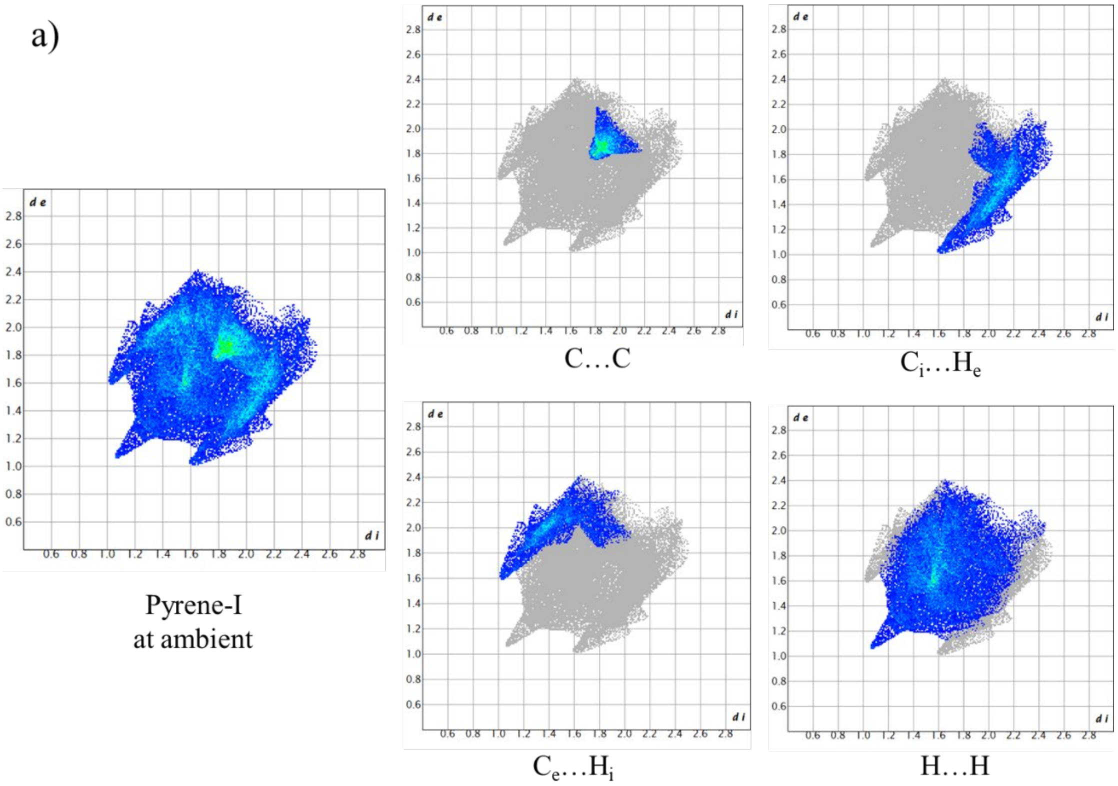

Pyrene-I
at ambient
C…C
C_i…H_e
C_e…H_i
H…H

b)

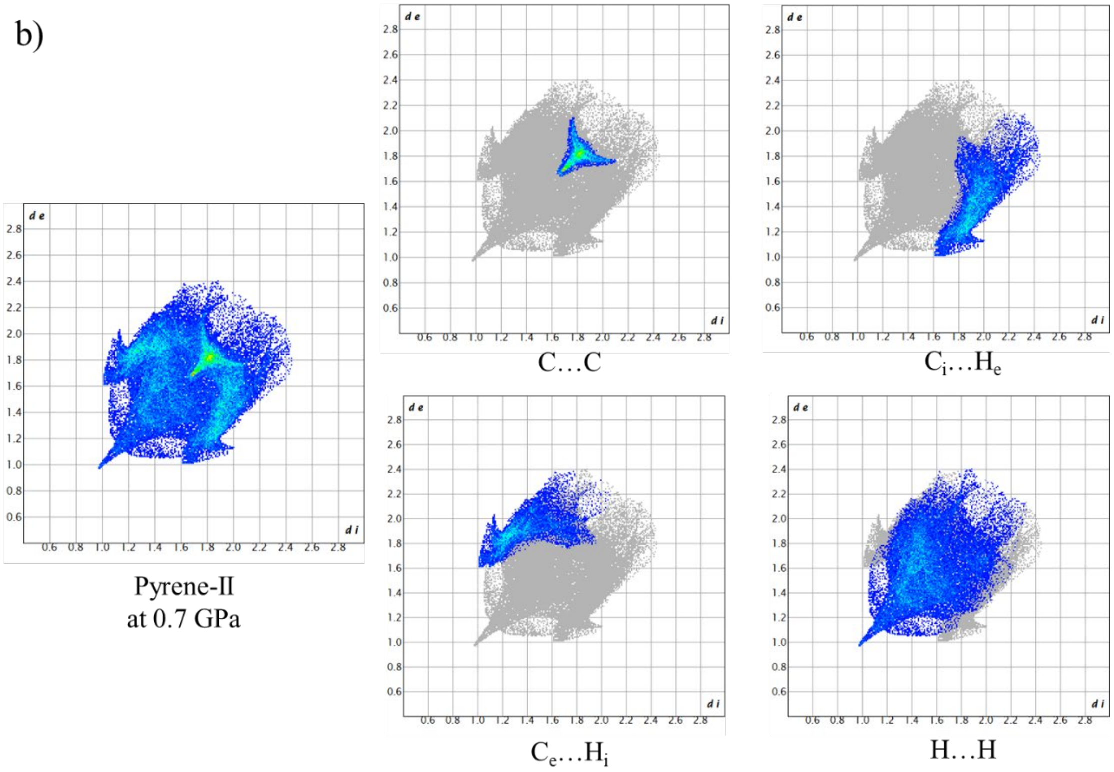

Pyrene-II
at 0.7 GPa
C…C
C_i…H_e
C_e…H_i
H…H

c)

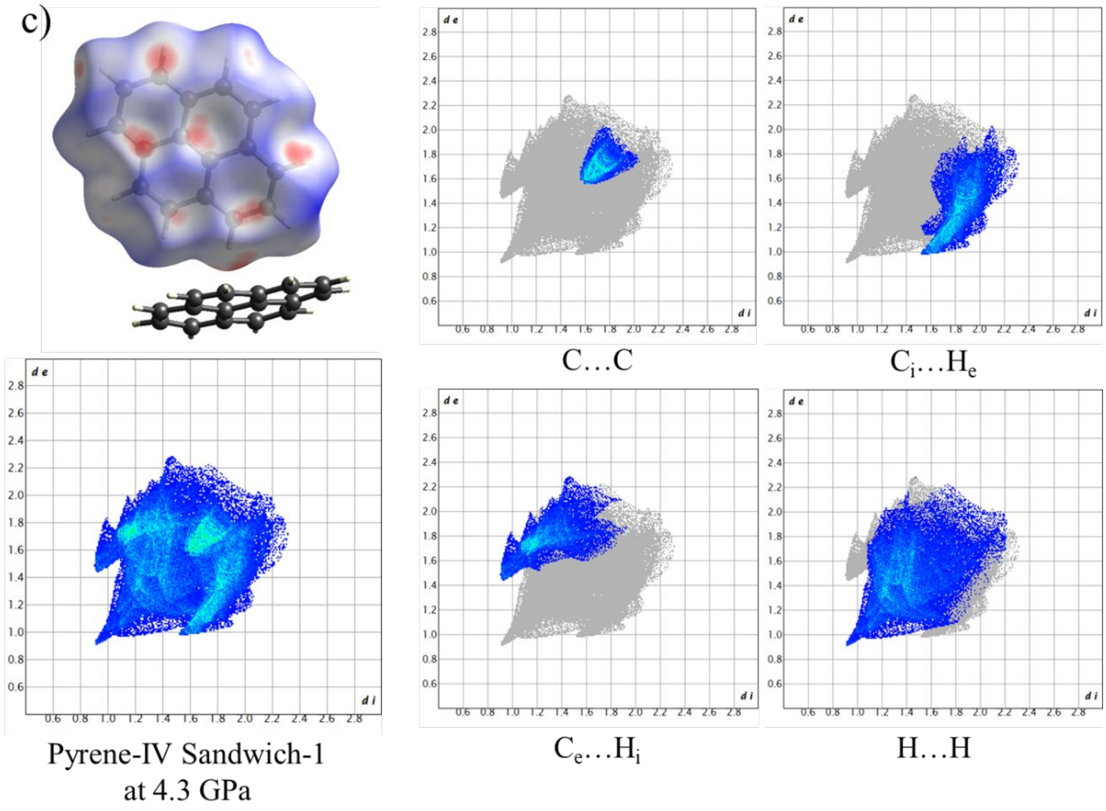
de
di
C…C
$C_i…H_e$
$C_e…H_i$
H…H
Pyrene-IV Sandwich-1
at 4.3 GPa

d)

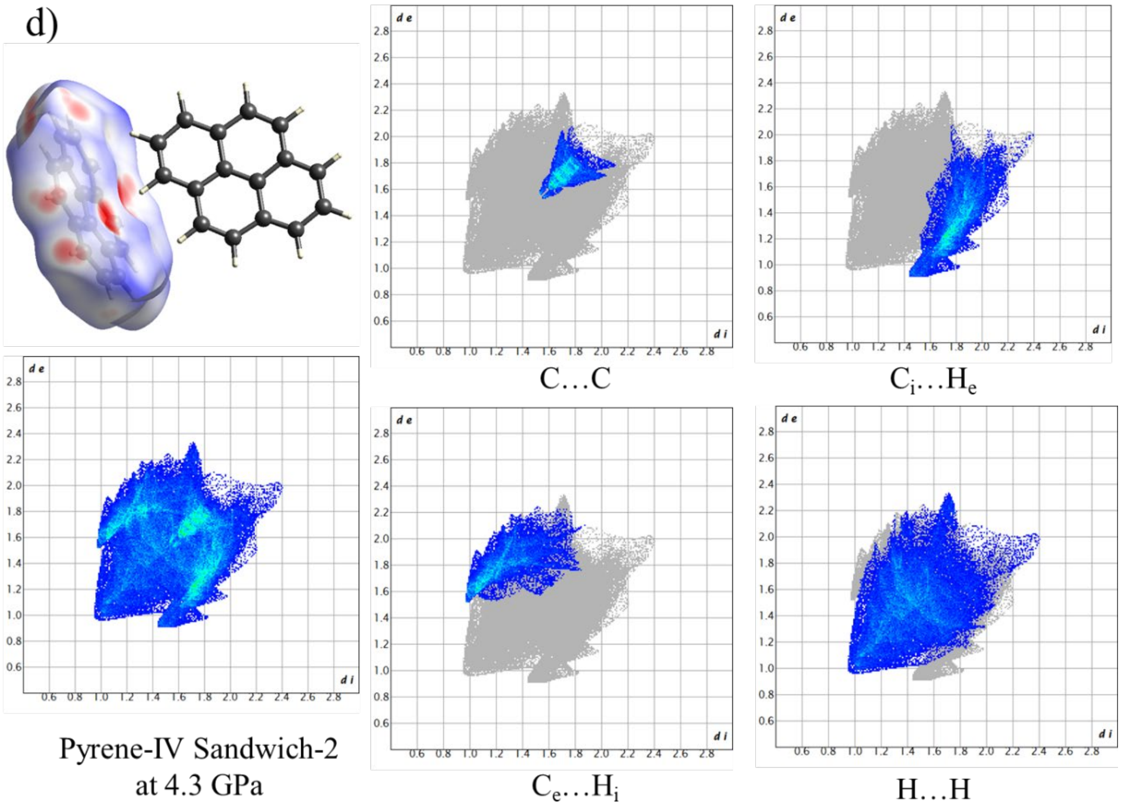
de
di
C…C
$C_i…H_e$
$C_e…H_i$
H…H
Pyrene-IV Sandwich-2
at 4.3 GPa

e)

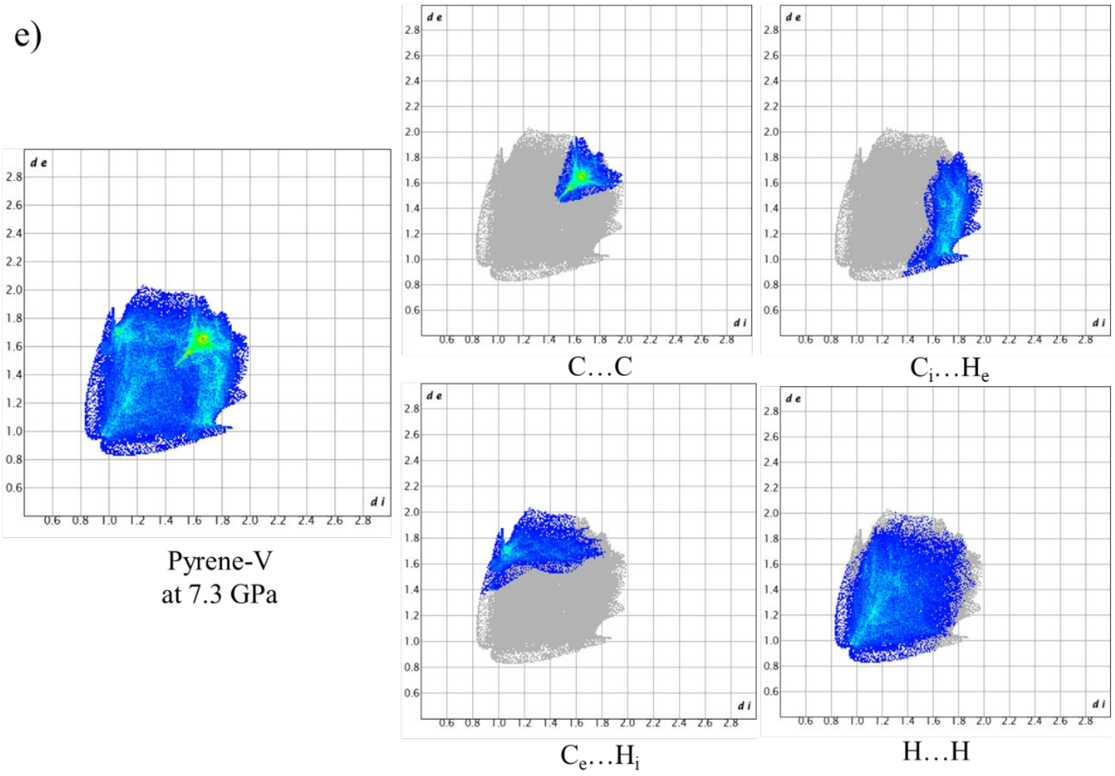

Pyrene-V
at 7.3 GPa
C…C
$C_i…H_e$
$C_e…H_i$
H…H

f)

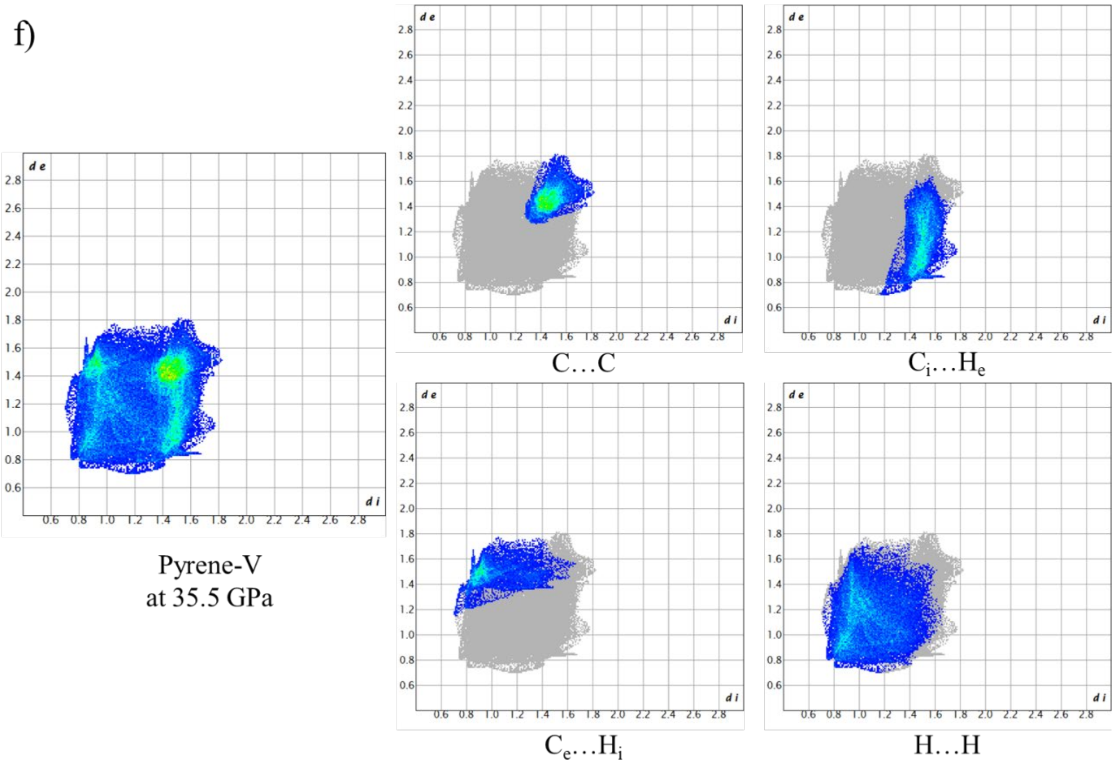

Pyrene-V
at 35.5 GPa
C…C
$C_i…H_e$
$C_e…H_i$
H…H

g)

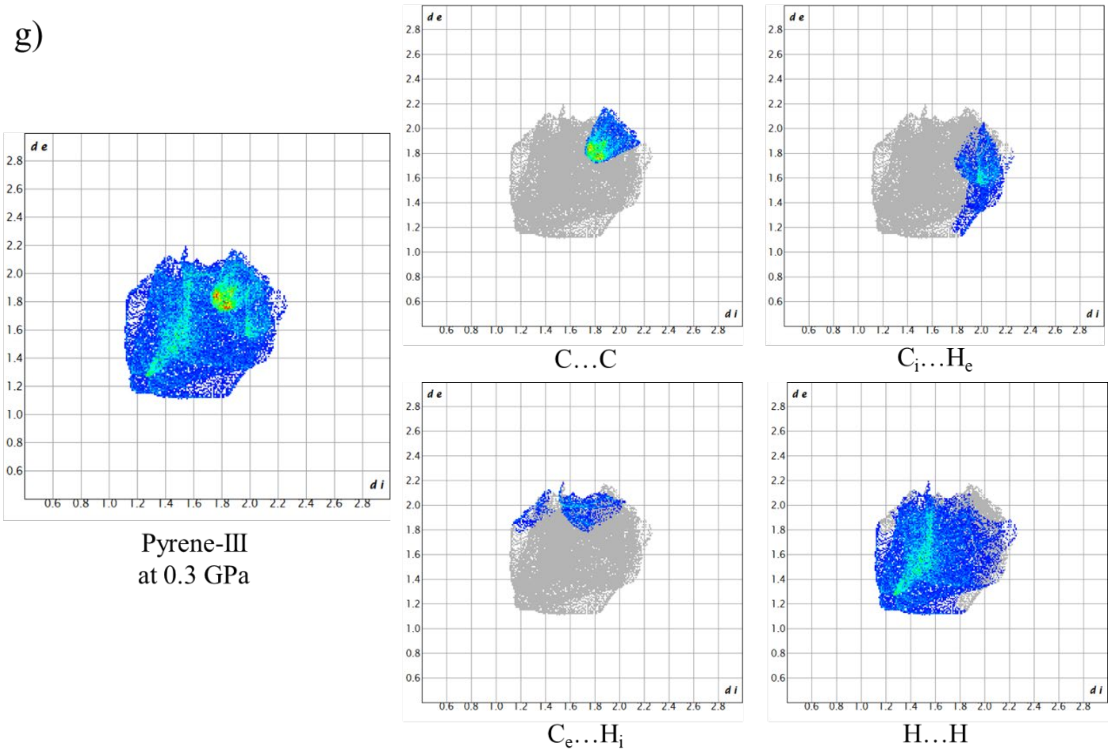

Supplementary Figure 2. Fingerplots for pyrene molecules in pyrene polymorphs at different pressures. Different intermolecular interactions are highlighted in separate plots. a) Pyrene-I at ambient conditions; b) pyrene-II at 0.7 GPa; c) pyrene-IV: a molecule in sandwich-1 at 4.3 GPa, the Hirschfeld surface mapped in d_{norm} is given for clarity; d) pyrene-IV: a molecule in sandwich-2 at 4.3 GPa, the Hirschfeld surface mapped in d_{norm} is given for clarity; e) pyrene-V at 7.3 GPa; f) pyrene-V at 35.5 GPa; g) pyrene-III at 0.3 GPa, the structure data are from ref. [6].
