# Supplementary material for: Polymorphism of pyrene on compression to 35 GPa in a diamond anvil cell
Source: Commun Chem. 2024 Sep 17;7:209. doi: 10.1038/s42004-024-01294-0 (PMC11405754; doi:10.1038/s42004-024-01294-0)
Supplement: Supplementary file 3 — Description of Additional Supplementary Files [file 42004_2024_1294_MOESM3_ESM.pdf]

Description of Additional Supplementary Files

File name: Supplementary Data 1 - 12

Description: Crystal structures of pyrene polymorphs. Full crystallographic and experimental data are provided in Supplementary Tables 2 through 5 and Supplementary Data 1 through 12.

File name: Supplementary Data 13

Description: Numerical source data for Figs 2, 3, 4, 5, 10, 12 are provided as separate tabs in one Excel file.
